# Long-Term Effects of Soil Remediation with Willow Short Rotation Coppice on Biogeographic Pattern of Microbial Functional Genes

**DOI:** 10.3390/microorganisms10010140

**Published:** 2022-01-11

**Authors:** Wenjing Liu, Kai Xue, Runpeng Hu, Jizhong Zhou, Joy D. Van Nostrand, Jannis Dimitrou, Laura Giagnoni, Giancarlo Renella

**Affiliations:** 1 College of Resources and Environment, University of Chinese Academy of Sciences, Beijing 101408, China; liuwenjing18@mails.ucas.ac.cn; 2 Beijing Yanshan Earth Critical Zone National Research Station, University of Chinese Academy of Sciences, Beijing 101408, China; 3 Key Laboratory of Adaptation and Evolution of Plateau Biota, Chinese Academy of Sciences, Xining 810001, China; 4 Department of Biological Sciences, Smith College, Northampton, MA 01063, USA; runpenghu@gmail.com; 5 Department of Microbiology and Plant Biology, Institute for Environmental Genomics, University of Oklahoma, Norman, OK 73019, USA; jzhou@ou.edu (J.Z.); joy.vannostrand@ou.edu (J.D.V.N.); 6 Department of Crop Production Ecology, Swedish University of Agricultural Sciences, 75007 Uppsala, Sweden; Jannis.Dimitriou@slu.se; 7 Department of Civil Engineering, Architecture, Environmental and Mathematics (DICATAM), University of Brescia, via Branze 43, 25123 Brescia, Italy; laura.giagnoni@unibs.it; 8 Department of Agronomy, Food, Natural Resources, Animals and the Environment, University of Padova, viale dell’Università 16, 35020 Legnaro, Italy

**Keywords:** functional gene diversity, short rotation coppice phytotechnology, soil microbial community, soil pollution, phytoremediation, biogeographic pattern

## Abstract

Short rotation coppice (SRC) is increasingly being adopted for bioenergy production, pollution remediation and land restoration. However, its long-term effects on soil microbial communities are poorly characterized. Here, we studied soil microbial functional genes and their biogeographic pattern under SRC with willow trees as compared to those under permanent grassland (C). GeoChip analysis showed a lower functional gene diversity in SRC than in C soil, whereas microbial ATP and respiration did not change. The SRC soil had lower relative abundances of microbial genes encoding for metal(-oid) resistance, antibiotic resistance and stress-related proteins. This indicates a more benign habitat under SRC for microbial communities after relieving heavy metal stress, consistent with the lower phytoavailability of some metals (i.e., As, Cd, Ni and Zn) and higher total organic carbon, NO_3_^−^-N and P concentrations. The microbial taxa–area relationship was valid in both soils, but the space turnover rate was higher under SRC within 0.125 m^2^, which was possibly linked to a more benign environment under SRC, whereas similar values were reached beyond thisarea. Overall, we concluded that SRC management can be considered as a phytotechnology that ameliorates the habitat for soil microorganisms, owing to TOC and nutrient enrichment on the long-term.

## 1. Introduction

Short rotation coppice (SRC) refers to the growth of multi-stemmed woody plants over two- to five-year rotation periods. In this cultivation practice, the plant roots are left intact in place and the nutrients are stored in the root system to ensure vigorous regrowth of the shoots for up to 30 years before the stand needs to be replanted. The species used in SRC are mostly willow (*Salix* spp.), poplar (*Populus* spp.) and their hybrids, eucalypt (*Eucalyptus* spp.) and black locust (*R*. *pseudoacacia*), because they are characterized by fast growth rates, high yields and are easily harvested. The first and largest SRC-managed areas were in Sweden, Poland, the USA and Northern Italy [1], but a steady increase in the number of SRC plantations worldwide has been favored by subsidies for more sustainable and integrated bioenergy supply chains [2]. Additional incentives to the conversion of agricultural land to SRC practices are related to its potential for C sequestration either in the soil or in woody biomass [3]. In fact, SRC has positive impacts on several soil properties, such as soil organic matter (SOM) and nutrient content, a reduction in soil compaction and erosion, and an increase in soil biodiversity [4], particularly on degraded soils [4,5].

Another application of SRC plantations is soil restoration by excessive nutrients or contaminants, in which fast-growing trees have proven to be effective, owing to their high biomass production and effective root uptake. In fact, willow and poplar SRC crops have been used in buffer strips to mitigate and prevent water eutrophication and to reduce heavy metal mobility in phytoremediation interventions on contaminated soils [6]. The treatment of contaminated urban and industrial effluents is typically conducted in treatment plants that remove potentially toxic elements and organic pollutants with high efficiency [7]. Although the sludge can be recycled as an amendment for agricultural soil, liquid effluents need remediation before being discharged into water bodies to prevent eutrophication and to maximize the recycling of nutrients through the valorization of the woody biomass.

Though SRC management is potentially beneficial to soil chemical and biological fertility [8], it is still a monoculture grown at high density. Its effects on the soil’s microbial functional genes, on their biogeographic pattern and on their microbial activity in the long term are still unclear. Unlike natural woodlands, SRC imposes a strong dominance in tree species. with a reduction in the complexity and heterogeneity of organic matter inputs, a parallel reduction in soil microbial diversity has been often postulated [9]. The effects of SRC management on soil microbial diversity and ecological functionality in the long term may be further aggravated by soil nutrient depletion and acidification due to intense tree growth [10].

Soil microorganisms play fundamental roles in terrestrial ecosystems, are involved in organic matter decomposition and nutrient mineralization and share ecological interactions with plants, producing biostimulants or acting as plant pathogens [11]. Changes in soil biochemical endpoints such as basal respiration, microbial biomass and enzyme activities can be taken as early indicators of the impact of a specific soil management [12], but the large degree of redundancy of microbial activities in soils may mask subtle yet important changes in the soil microbial communities. In soils under SRC, due to the combined effects of a lack of tillage and litter accumulation, no reduction in soil respiration or soil enzyme activities has been reported, and an altered microbial community structure, as compared to forest or grassland, has been observed, with the current knowledge mostly having been provided by short- and medium-term (2 to 8 years) field observations, whereas the information on the long-term effects of on soil microorganism of SRC plantations used for phytoremediation is still scant and/or controversial.

In-depth information on the functional diversity of soil microbial communities can be obtained through metagenomic approaches [13,14]. Among the various currently available molecular techniques to analyze the functional diversity of soil microbial communities, the GeoChip microarray [15] can detect up to 10 [4] genes from over 400 gene categories belonging to microbial groups involved in key soil functions, such as nutrient cycles, the degradation of organic contaminants, metal resistance and ecological interactions. The GeoChip technique has been used to assess the impact of landfill leachate on the functional diversity of microbial communities [16], and the positive effects of phytomanagement with willow SRC on the microbial functional diversity of heavy-metal-contaminated soils [17,18,19].

Althougb field trials have demonstrated that willow plants can remove various pollutants from effluents from water treatment plants [20], the long-term effects of this practice on soil microbial functional communities are still poorly documented. We hypothesized that long-term treatment of effluents from a water treatment plant with willow SRC management would not significantly alter the soil functional activity or functional diversity. We tested our hypothesis by analyzing the chemical properties, microbial functional gene diversity, microbial biomass and various enzymatic activities of a soil with willow trees or mixed grassland after 9 y of effluent treatment. Microbiological and biochemical endpoints were correlated with the total concentration and availability of nutrients and heavy metals.

## 2. Materials and Methods

### 2.1. Site Characteristics and Soil Sampling

The experimental site was located in Högbytorp, Sweden, and consisted of a long-term field trial established in 2004 on a previous arable clay soil with an average clay content of 38%, adjacent to an active landfill operated by Ragnsells Avfallsbehandling AB (Upplands-Bro, Sweden) as described by Aronsson et al. (2010) [20]. In 2005, cuttings of Tora (*Salix schweinii* × *Salix viminalis*) and Gudrun (*Salix dasyclados*) clones were planted in a double-row scheme of 400 m^2^ square plots, and two replicate plots were prepared for each willow clone. The leachate generated by the landfill and contaminated mainly by Cd, Cr and Zn was conveyed to both the willow SRC plots and to an adjacent grassland considered as a control area by means of an irrigation system. The original field trial was established on a soil comprising only two control (C) and two willow SRC plots receiving the landfill leachate [20]. Table 1 shows the main soil chemical properties and total metal concentrations of the studied soils.

To minimize the variability of microbial functional activity and functional gene diversity due to under-sampling, unequal sampling and taxonomic lumping due to the presence of two field replicate plots per treatment in the experimental design [21,22], we used the protocol for soil sampling proposed by Zhou et al. (2008) [23]. To provide a typical and representative sample of the diversity at the meter scale of interest, 11 samples were taken from each of the two replicate C and SRC plots for a total of 22 samples per treatment. The auger was thoroughly cleaned between each soil core sampling, and the polyethene bags were new and were sealed after the placement of the soil cores, and all plastics and glassware used for preparing the soil samples for DNA extraction were sterile. Each of the twenty-two samples was kept separated and split into subsamples, stored at −80 °C for DNA extraction, at 4 °C for biochemical analyses and air-dried for chemical analyses.

### 2.2. Analysis of Soil Chemical Properties and Soil Toxicity

Using 0.5 g of dry soil suspended in 10 mL of concentrated HNO3 and stepwise heated up to 170 °C for 10 min followed by 6 min at 180 °C, the quantification of elemental pseudototal concentration was performed via soil microwave assisted digestion (Ethos1, Milestone, FKV, Italy) according to the US EPA (2007) method. Blanks and reference material (ERM CC141) were also analyzed for each batch to assess eventual metal contamination and if the extraction recovery was in the range 85–110%. The availability of heavy metals was evaluated through extraction with 1M NH_4_NO_3_ (ISO 19730:2008) [24] buffered at pH 7.00 with concentrated ammonia. An ICP optical spectrometer (Vista MPX, Varian, Australia) was used for the quantification of both elemental pseudo-total concentrations and availability. The total organic C (TOC) was measured using the method of Walkley and Black (1934) [25], and the total N concentration was determined using a CHN-S Flash E1112 elemental analyzer (Thermo Finnigan) with the standard method (ISO 10694:1995) [26]. Inorganic N (NH_4_^+^-N and NO_3_^−^-N) was extracted by shaking 5 g soil for 1 h with 1M KCl (1:5 soil:solution ratio) according to Keeney and Nelson (1982) [27] and quantified using ion-selective electrodes (Crison IES, Spain). The total soil P concentration was extracted using the wet digestion method [28], organic P was extracted with the method of (Bowman and Moir, 2007) [29], plant available P was extracted using the method of Bray and Kurz (1945) [30] and concentrations of both P fractions were quantified via UV spectrophotometry at 880 nm after reaction of the extracts with the sulfo-molybdic acid reagent (Murphy and Riley, 1962) [31].

Soil toxicity was evaluated using the BioTox test (Aboatox, Turku, Finland) based on the bioluminescence inhibition of *Vibrio fischeri*, and the soil was considered toxic for bioluminescence inhibition >20%.

### 2.3. Analysis of Soil Microbial Biomass, Soil Respiration, N Mineralization Potential and Soil Enzyme Activities

The soil respiration rate was quantified by gas chromatography (HP 5890) to measure the CO_2_-C evolution [32]. Soil microbial biomass was estimated based on the content of ATP according to Ciardi and Nannipieri (1990) [33]. Soil N mineralization was determined by incubating 10 g of moist soil in conical sealed flasks for 28 d, aerated every 7 d. Concentrations of NH_4_^+^-N and NO_3_^−^-N were determined prior to and at the end of incubation through extractions with 0.02 M KCl using ion-selective ion electrodes (Crison IES, Spain). The N mineralization potential was analyzed by determining ammonification and nitrification potentials, calculated based on the difference between the concentration of NH_4_^+^-N and NO_3_^−^-N concentrations prior to and after 28 d of incubation at 50% of water-holding capacity and 25 °C in the dark. The method of Tabatabai and Bremner (1969) [34] was used to observe alkaline and acid phosphomonoesterase activities, the phosphodiesterase activity was determined with the method of Browman and Tabatabai (1978) [35], the arylesterase activity was determined with the method of Zornoza et al. (2009) [36]. The β-glucosidase and β-galactosidase activities were determined using the protocol described by Tabatabai (1982) [37]. The urease activity was determined according to Nannipieri et al. (1974) [38], and the protease activity was determined using N-benzoyl-amide as a substrate (Ladd and Butler, 1972) [39].

### 2.4. Functional Gene Diversity via GeoChip Analysis

The abundance and diversity of microbial functional genes were analyzed using GeoChip 4.2 on DNA extracted by freeze-grinding mechanical lysis. The Geochip contained 107,950 probes, covering 792 functional gene families from 11 major functional categories, including C, N, P and S cycling categories, plus metal and antibiotic resistance genes [15]. Full details on the DNA labeling, hybridization, image analysis and data processing were reported by Xue et al. (2015, 2018) [17,18,19].

### 2.5. Data Analysis

The soil chemical and biochemical data were analyzed via ANOVA, followed by the Fisher LSD test for sample comparisons. Using GeoChip technology, the functional gene diversity of the microbial communities in Unt and SRC soils, assessed using the richness (detected probe number), Shannon–Weaver (H) and Simpson Reciprocal indices, was compared using a *t*-test. The Shannon–Weaver index is defined as *H* = −∑*p_i_* × *ln*(*p_i_*), where *pi* is the proportional abundance of species *i*. Simpson’s index is based on *D = ∑p_i_*^2^. The richness was represented by the number of detected probes. For the calculation of diversity indices, the detected gene probes were considered as species and their abundances were represented by the normalized signal intensities. Changes in functional gene composition under Unt and SRC management were assessed by means of detrended correspondence analysis (DCA) and non-parametric similarity tests—the multiple response permutation procedure (MRPP), permutational multivariate analysis of variance (Adonis) and analysis of similarity (ANOSIM) based on the Bray–Curtis dissimilarity index. Analysis of variance (ANOVA) was used to compare the normalized functional gene abundances, with individual probes included as a factor to partition the variance from various probes within each gene catalogue. The link between microbial functional gene composition and soil properties was assessed using the Mantel test. All statistics and modeling were performed using R version 3.0.2 (The R Foundation for Statistical Computing, Vienna, Austria) and significant differences were defined as *p* < 0.05.

## 3. Results

### 3.1. Soil Properties, Elemental Concentration and Availability and Soil Toxicity

The C and SRC soils showed similar pH values and total N contents, whereas the SRC soil had significantly higher TOC, total P, soluble P, organic P and NO_3_^−^-N, but significantly lower NH_4_^+^-N and available P concentrations than C soil (Table 1).

Among the measured elements, the SRC soil presented higher total concentrations of Cd, Cu and Mn, Na, Ca and K, and the C soil presented higher total concentrations of Cr, Ni, Zn, Mg and Fe, whereas the total Al, As and Pb concentrations were not significantly different between the two soils (Table 2). The SRC soils presented higher Al, Cr, Cu, Pb, Na, K and Mg availability, whereas the C soil presented higher As, Cd, Ni, Zn, Ca and Fe availability (Table 2). Bioluminescence inhibition, determined using the BioTox test, was below 20% for both the SRC and C soils, indicating the lack of toxicity of both soils. Total concentrations of K, Mg and Fe were higher in the C soil than in SRC soil, whereas the corresponding NH_4_NO_3_-exchangeable concentrations were lower in the C soil than in SRC soil (Table 2). Concentrations of pseudo-total and exchangeable Na were higher in SRC than in C soil, whereas the pseudo-total and exchangeable concentrations of Ca were higher in the C soil than in the SRC soil (Table 2).

### 3.2. Soil Microbial Biomass and Biochemical Activity

**Figure 1:**
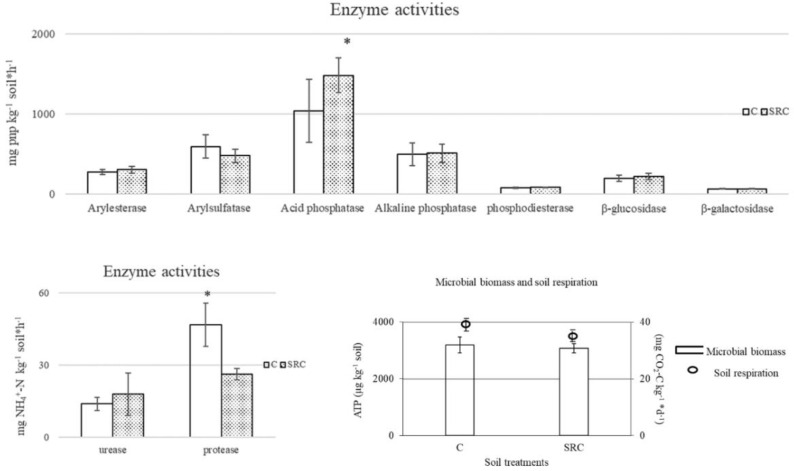
Enzyme activities, microbial biomass and respiration of SRC and C soils. The symbol * indicates significant differences (*p* < 0.05) between values of C and SRC soils for each biochemical parameter.

Enzyme activities showed similar values in both C and SRC soils, with the exception of higher acid phosphomonoesterase activity in the SRC soil and higher protease activity in the C soil (Figure 1). The C and SRC soils showed similar values of soil microbial biomass and respiration rates (Figure 1).

The nitrification rates (mg NO_3_^−^-N kg^−1^ d^−1^) of C and SRC were 1.086 and 0.82, respectively, whereas the ammonification rates (mg NH_4_^+^-N kg^−1^ d^−1^) were 34.5 and 31.8, respectively, and the differences between C and SRC soils were significant (*p* < 0.01) in both cases.

### 3.3. Diversity and Composition of Soil Microbial Functional Communities

**Figure 2:**
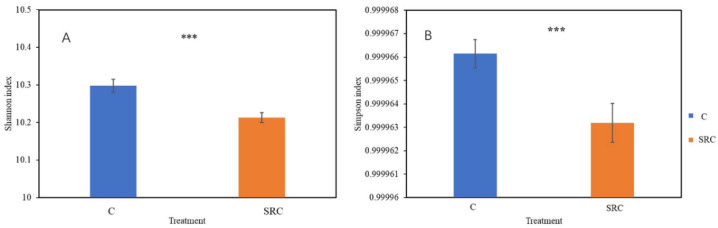
Diversity represented by Shannon (**A**) and Simpson (**B**) indexes. Error bars represent the standard error (*n* = 22). *** = significant at *p* < 0.001.

**Figure 3:**
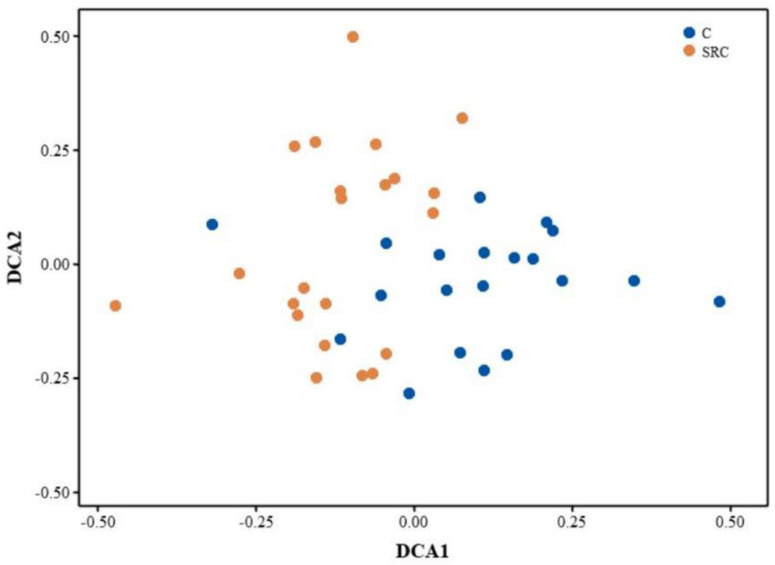
DCA profile for functional genes from soil microbial community in C (blue) and SRC (orange) soils.

A total of 37,363 gene probes were detected by GeoChip across all samples, of which 37,236 and 37,354 were in the SRC and C soils, respectively. The alpha diversity for the microbial functional community, represented by the Shannon or Simpson indices, was significantly lower in the SRC soil than in the C soil (Figure 2). The DCA profile showed that the microbial functional community of the SRC soil clearly differed from that of the C soil (Figure 3). MRPP, ANOSIM and ADONIS dissimilarity tests confirmed the significant differences (*p* ≤ 0.001) in microbial functional gene composition between the SRC and C soils (Table 3).

### 3.4. Taxa–Area Relationship for Microbial Functional Genes

**Figure 4:**
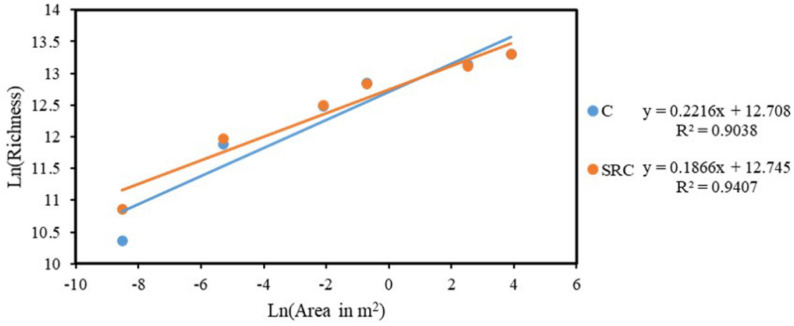
Taxa–area relationships of functional genes of in C (blue) and SRC (orange) soils. The taxa-area relationship was fitted by means of linear regression in the ln-transformed richness and the ln-transformed area of six nested areas (0.0002, 0.005, 0.125, 0.5,12.5 and 50 m^2^).

The taxa–area relationship (TAR) was tested for microbial functional communities in SRC and C soils and the linear regression between the ln-transformed richness for microbial functional gene community over the ln-transformed area (m^2^) is shown in Figure 4. Represented by the slope in the linear regression, the space turnover rate for microbial functional communities in SRC soil (z = 0.1866) was higher than that in C soil (z = 0.2216). Notably, before reaching the area of 0.125 m^2^, the overall richness of the microbial functional communities in the SRC soil was higher than that of those in the C soil, e.g., accounting for 158,198 and 145,064 OTUs, respectively, when the area reached 0.125 m^2^. Beyond the scale of 0.125 m^2^, the richness of the microbial functional communities of C and SRC soils were similar, ranging from 262,880 to 601,928 (Figure 4).

### 3.5. Functional Genes for Stress, Metal and Antibiotic Resistance

**Figure 5:**
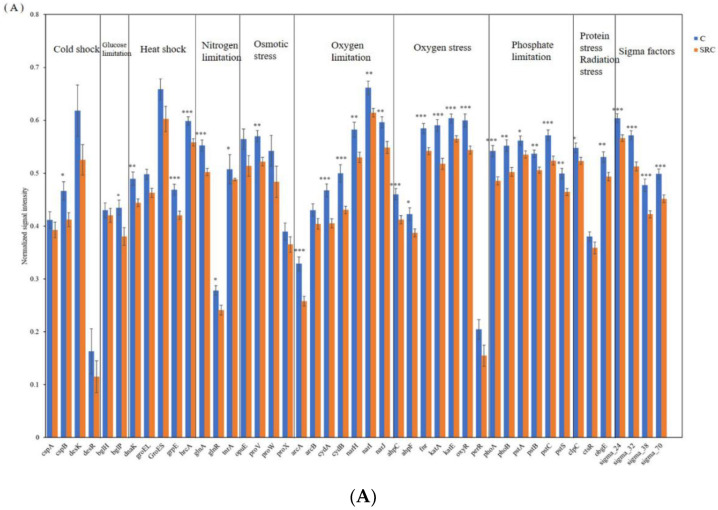

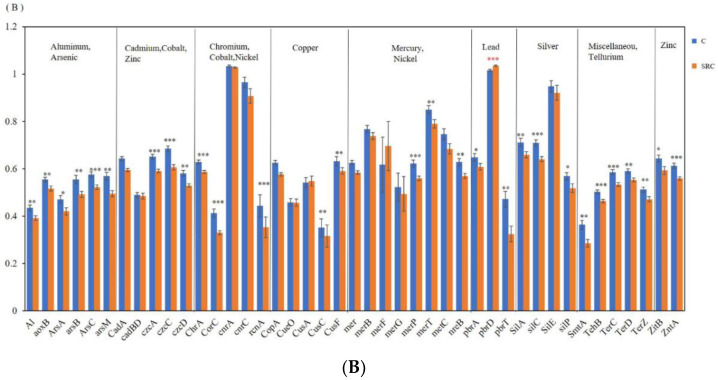
Abundance of functional genes encoding for stress (**A**) and for metal resistance (**B**) in the C (blue) and SRC (orange) soils. Error bars represent standard error. * indicates *p*-value ≤ 0.05; ** = significant at *p*-values ≤ 0.01; *** = significant at *p*-values ≤ 0.001. The * symbols in black indicate that untreated soil (C) > *Salix*-treated soil (SRC) and * symbols in red indicate that untreated soil (C) > *Salix*-treated soil (SRC).

Thirty-three of the 45 microbial functional genes responsible for microbial reactions to various stresses had generally significantly lower abundances (*p* < 0.05) in SRC than in C soil (Figure 5A), including cspB, dnaK, grpE and hrcA encoding for cold shock protein; bgIP encoding for glucose limitation; proV encoding for osmotic stress; clpC encoding for protein stress and obgE encoding for radiation stress; six out of seven oxygen stress genes (ahpC, ahpF, fnr, katA, katE, oxyR); six of seven oxygen limitation genes (arcA, cydA, cydB, narH, narI, narJ); all N limitation genes (gllnA, glnR, tnrA); all P limitation genes (phoA, phoB, pstA, pstB, pstC, pstS); and all Sigma factors genes (sigma_24, sigma_32, sigma_38, sigma_24), whereas the other detected stress genes did not differ between soil treatments (Figure 5A).

Forty-four functional genes involved in metal(-oid) resistance were detected in all analyzed soils (Figure 5B). Except for the abundance of pbrD, one of the three Pb-resistance genes that was significantly (*p* < 0.001) higher in the SRC that in C soil, most functional genes for metal(-oid) resistance had lower abundances in the SRC soils than in the C soils (*p* < 0.05). Among them, abundances of all As-resistance genes (aoxB, arsB, ArsA, ArsC and arsM); all metal(-oid) resistance genes (TehB, TerC, TerD, TerZ) encoding cation efflux system proteins for Cd, Co and Zn resistance (czcA, czcC and czcD); genes encoding cation transport ATPase for Zn resistance (ZitB and ZntA); ChrA encoding chromate ion transporter protein for Cr resistance; CorC encoding Mg- and Co- efflux protein for Mg and Co resistance; rcnA encoding Co and Ni resistance; and nreB encoding an Ni-induced transporter for Ni resistance were significantly higher (*p* < 0.05) in C soil than in SRC soil. Similarly, two of the three Pb resistance genes (pbrA and pbrT), two of the five Cu resistance genes (CusA and CusF), two of seven genes encoding mercuric transport proteins for Hg resistance (merP and merT), three of four genes encoding outer membrane cation efflux protein for Ag resistance (silA, silC and silP), and SmtA encoding metallothionein resistance to various metals had significantly lower (*p* < 0.05) abundances in SRC than in C soils. The abundance of the other detected of metal(oid) resistance genes did not differ between treatments (Figure 5B).

**Figure 6:**
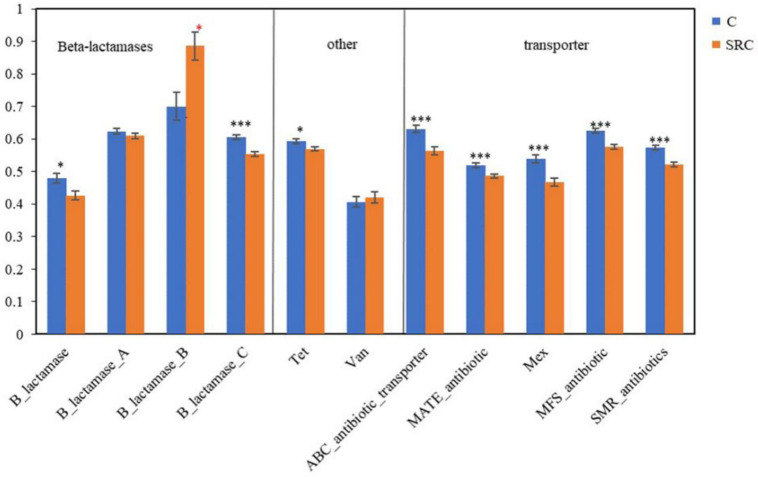
Abundance of functional genes encoding antibiotic resistance in the C (blue) and SRC (orange) soils. Error bars represent standard error. Symbols * and *** indicate significant differences at *p*-values ≤ 0.05 and ≤ 0.001, respectively. The * symbols in black indicate C > SRC, and * symbols in red indicate C > SRC.

Among the functional genes conferring antibiotic resistance, eight out of 11 had significantly lower abundances (*p* < 0.05) in SRC soils than in the C soils, including all transporter genes, and the gene encoding class C of b-lactamases and Tet for the methylcytosine dioxygenase, whereas the gene encoding class B of b-lactamases had significantly (*p* < 0.05) higher abundance in the SRC than in the C soils (Figure 6).

### 3.6. Linkage of Microbial Functional Composition with Soil Properties or Biochemical Activity

The Mantel test was performed to determine the linkage between microbial functional composition and soil properties (Table S1). For all samples, microbial functional community composition was significantly correlated with soil properties such as ammonia (*p* < 0.05); Soluble P (*p* < 0.01); Olsen P (*p* = 0.001); total concentrations of As, Cd, Cr, Cu, Mn, Zn, Na, Ca and K and the availability of Al, As, Cd, Cu, Mn, Pb, Zn, Ca, Mg and Fe (*p* < 0.05). Moreover, the nitrification potential and CO_2_-C evolution were also correlated with the microbial functional community composition. Among enzyme activities, the microbial functional community was only correlated with protease activity (Table S1).

In the C soils, microbial functional community composition was significantly correlated to total P (*p* < 0.05), soluble P (*p* < 0.01), total K (*p* < 0.05) and total Cd concentration (*p* < 0.05). In the SRC soil, the microbial functional community composition was correlated with the pH value (*p* < 0.01), total Zn (*p* < 0.05), available Ni (*p* < 0.05) and available Pb (*p* < 0.05) concentrations. Regarding the measured enzyme activities, the microbial functional community composition was only correlated with β-galactosidase activity (*p* < 0.05) in the SRC soils.

## 4. Discussion

The higher TOC, total N and total and organic P contents of the SRC soils as compared to the C soils have confirmed the previous findings on the potential of this management with woody plants to store C and enrich soil with nutrients [40,41], and the 67% TOC increase in SRC soil as compared to C soil (Table 1) represents a considerable long-term C accumulation related to the soil conversion to SRC. The significantly higher nitrification and ammonification rates could be considered as a confirmation of the faster SOM mineralization rates in the C as compared to the SRC soils [42], and could be related to its higher protease activity, which catalyzes the release of aminoacids from proteins and peptides. The significantly lower available P and Fe concentrations (Table 1) could be considered signs of the willows’ high nutrient demand, but the higher total N, P and K contents of the SRC over the C indicated that globally the SRC management led to positive nutrient balance in the long term. The increase in the C/N ratio of the SRC as compared to the C soil could be ascribed to the slow litter degradation of the lignin in the willow litter [43] and to the high root exudate release and fast turnover of fine roots [44], all factors that are likely contributed to the increase in soil TOC. Owing to its fundamental ecological functions as a regulator of the cation exchange capacity, buffering the soil pH value and as a reservoir of C and other primary nutrients, soil TOC is an integrative indicator of soil quality [45].

Most of the soil enzyme activities responsible for SOM decomposition and C, N, P and S mineralization were not significantly different between C and SRC soils, indicating no impacts on the potential microbial functional activity [46] or links to the high variability commonly observed in enzyme activity measurements [47]. These results parallel the values of microbial biomass and soil respiration rates, and indicate that, on the long term, the SRC regime does not reduce the potential microbial mineralization capacity [48]. The significantly higher acid phosphomonoesterase activity in the SRC than in the C soil could be ascribed to the higher organic P content the SRC soils, as well as to the release of acid phosphatase by the willow roots [49], and could be responsible for the higher P solubility in the SRC soils (Table 1). The lower protease activity in SRC soil has been previously reported [50].

The microbial diversity based on functional genes was significantly lower in soils under SRC than in the C soils, which is consistent with previous studies by Xue et al. (2020) [18] also using GeoChip and by Foulon et al. (2016) [51] using 16S rRNA for taxonomic information. However, increased diversity of functional genes has also been reported in SRC soils [17,19]. These conflicting results might be explained by different phytoremediation technologies (mixed grassland vs. willow or poplar), toxicity types and levels and the effectiveness of remediation.

Soils under SRC management showed a reduced space turnover rate of soil microbial functional communities. The taxa–area relationship (TAR), referring to the general increase in species richness with area, is one of the most commonly used biogeographic patterns for the analysis of diversity and distribution patterns at multiple spatial scales [52]. The space turnover rate in TAR for microbial spatial scaling has been reported to vary substantially with temperature [53], long-term fertilization [54], land use [55] and degradation gradients [56]. The decreased space turnover rate of soil microbial functional communities found in this study was likely linked to the reduced stress for microbial communities in SRC soils. The reduced stress for microbial communities was evidenced by decreased abundances of microbial functional genes involved in stress, which could be ascribed to the TOC accumulation as compared to the C soil, as microbial activity in soil is generally limited by carbon availability [57]. The lower availability of As, Cd, Ni and Zn in the SRC as compared to the C soil could be another important factor in stress relief for soil microorganisms [17,58]. The lower space turnover rates have already been previously reported in relative benign environments, e.g., restored grasslands [59] and undegraded alpine meadow [55], probably because changes in soil physicochemical properties induce changes in the soil bacterial community [60]. To our knowledge, this is the first study showing the long-term effects of SRC management on the microbial space turnover rate, and future research could focus on a finer assessment of the impact of the SRC management main factors such as plant clone, plant density, coppice cycle and soil type on the long-term.

The microbial functional genes conferring metal resistance in SRC had lower abundances as compared to C soils. Similarly, lower abundances of microbial functional genes encoding for antibiotic resistance in SRC soils was also observed. Consistently, previous studies also found lower abundances of most functional genes encoding for antibiotic resistance in SRC soils than in untreated soils. As mentioned [18], this phenomenon could be explained by the fact that microbial genes encoding for metal resistance and antibiotic resistance are usually on the same mobile genetic element [61]. Genetic linkage between antibiotic resistance genes and metal resistance genes in microorganisms has also been reported [62].

The composition of microbial functional communities was significantly correlated with the total Cd concentration in the C soils, indicating that Cd pollution created great stress for soil microbial communities. In contrast, when Cd availability decreased to 0.04 mg kg^−1^ in the SRC soils, the total Cd concentration was no longer correlated with the microbial functional community, implying the relief of Cd stress. Interestingly, though pH did not significantly differ between SRC and C soils, it became an important factor for the shaping of the microbial functional community in C soils only, likely indicating that its importance increased when Cd stress was relieved. However, the microbial functional communities in SRC soils were also linked to Zn, despite its lower concentration in SRC soils, also indicating its importance when Cd stress was relieved.

## 5. Conclusions

All measured chemical and biochemical indicators for soil fertility showed that long-term SRC management increased soil TOC and reduced the availability of some metals (i.e., As, Cd, Ni and Zn) as compared to the same soil under grassland management, with both receiving a landfill leachate. Analysis of microbial functional genes showed lower diversity and lower relative abundances of microbial genes encoding for metal(-oid) resistance, antibiotic resistance and stress-related proteins under SRC, indicating an improved soil habitat that was more benign for the microbial community. The space turnover rate in the microbial taxa–area relationship was higher under SRC within 0.125 m^2^, possibly linked to a more benign environment under SRC as well. Thus, SRC management can be considered as a phytotechnology to ameliorate the habitat for soil microorganisms owing to TOC and nutrient enrichments in the long term, which would be useful to further optimize productive soil use.

## Figures and Tables

**Table 1:** Main soil chemical proprieties of SRC and C soils. Values are the means (*n* = 22) and the standard deviation is reported in brackets. Different superscripts indicate significant differences at *p* < 0.05.

	pH	TOC (g kg^−1^)	Total N (g kg^−1^)	NH_4_^+^-N (mg kg^−1^)	NO_3_^−^-N (mg kg^−1^)	Total P (mg kg^−1^)	Available P (mg kg^−1^)	Soluble P (mg kg^−1^)	Organic P (mg kg^−1^)
C	6.79 ^a^ (0.88)	14.3 ^a^ (1.42)	1.87 ^a^ (0.25)	**0.29**^a^ (0.03)	0.59 ^b^ (0.09)	190 ^b^ (15.3)	**32.7**^a^ (3.28)	0.27 ^b^ (0.02)	124 ^b^ (14.6)
SRC	6.47 ^a^ (0.71)	23.9 ^a^ (2.14) ^a^	1.96 ^a^ (0.31)	0.20 ^b^ (0.03)	**0.84**^a^ (0.08)	**286**^a^ (39)	19.0 ^b^ (2.44)	**0.48**^a^ (0.05)	**190**^a^ (20.9)

**Table 2:** Pseudo-total and exchangeable elemental concentrations in SRC and C soils. Values are the means (*n* = 22) and the standard deviation is reported in brackets. Different superscripts indicate significant differences at *p* < 0.05.

Soils		Pseudototal Elemental Concentration (mg kg^−1^)												
	Al	As	Cd	Cr	Cu	Mn	Ni	Pb	Zn	Na	Ca	K	Mg	Fe
C	30,943 ^a^ (1931)	9.21 ^a^ (1.96)	0.26 ^b^ (0.07)	**36.7** ^a^ (4.34)	46.1 ^b^ (7.85)	294 ^b^ (36)	**35.6** ^a^ (4.31)	17.6 ^a^ (1.74)	**147** ^a^ (25.7)	434 ^b^ (58)	8412 ^b^ (4020)	6562 ^b^ (1239)	**8619** ^a^ (493)	**33,052** ^a^ (3870)
SRC	30,531 ^a^ (7822)	8.98 ^a^ (1.08)	**0.37** ^a^ (0.17)	25.0 ^b^ (4.35)	**52.9** ^a^ (8.12)	**493** ^a^ (39)	32.1 ^b^ (3.73)	19.3 ^a^ (3.29)	83.6 ^b^ (6.84)	**635** ^a^ (86)	**11,606** ^a^ (1845)	**8210** ^a^ (756)	7282 ^b^ (931)	27,921 ^b^ (10229)
		**Elemental Availability (mg kg^−1^)**												
	Al	As	Cd	Cr	Cu	Mn	Ni	Pb	Zn	Na	Ca	K	Mg	Fe
C	0.16 ^b^ (0.04)	**0.23** ^a^ (0.07)	**0.07** ^a^ (0.01)	0.03 ^b^ (0.01)	0.08 ^b^ (0.02)	0.09 ^b^ (0.02)	**0.22** ^b^ (0.07)	0.03 ^b^ (0.01)	**0.09** ^a^ (0.02)	34.7 ^b^ (3.97)	**240** ^a^ (3.32)	264 ^b^ (3.64)	206 ^b^ (2.84)	**65.3** ^a^ (5.33)
SRC	**3.95** ^a^ (0.11)	0.07 ^b^ (0.02)	0.04 ^b^ (0.01)	**0.06** ^a^ (0.02)	**0.18** ^a^ (0.04)	**0.24** ^a^ (0.04)	0.09 ^a^ (0.02)	**0.09** ^a^ (0.01)	0.05 ^b^ (0.01)	**40.1** ^a^ (7.35)	103 ^b^ (2.06)	**303** ^a^ (6.08)	**390** ^a^ (7.82)	48.1 ^b^ (4.12)

**Table 3:** Non-parametric analyses to test the dissimilarity of communities between C (R) and SRC (S) soils. All three tests were multivariate analyses based on Bray–Curtis, Horn and Euclidean dissimilarity indexes.

C vs. SRC	MRPP ^1^		ANOSIM ^2^		ADONIS ^3^	
	δ	*p* ^4^	R	*p*	F	*p*
Bray–Curtis	0.15668	0.001	0.2745	0.001	3.7405	0.001
Horn	0.141183	0.001	0.2662	0.001	3.7746	0.001
Euclidean	89.38772	0.001	0.319	0.001	2.5449	0.001

^1^ Multi-response permutation procedure. δ is the overall weighted mean of within-group means of the pairwise dissimilarities among sampling units. The significance test is the fraction of permuted deltas that are less than the observed delta values. ^2^ Analysis of similarities. R is based on the difference of mean ranks between groups and within groups. The significance of the observed R is assessed by permuting the grouping vector to obtain the empirical distribution of R under the null model. ^3^ Permutational multivariate analysis of variance using distance matrices. Significance tests were performed by means of F-tests based on sequential sums of squares from permutations of the raw data. ^4^ *p*-values from corresponding significance tests.

## Data Availability

Not applicable.

## Supplementary Materials

The following supporting information can be downloaded at: https://www.mdpi.com/article/10.3390/microorganisms10010140/s1, Table S1: Mantel tests for correlations between microbial functional genes and soil chemical properties, or biochemical activities in untreated and Salix-treated soils.
