# Supplementary material for: Long-Term Effects of Soil Remediation with Willow Short Rotation Coppice on Biogeographic Pattern of Microbial Functional Genes
Source: Microorganisms. 2022 Jan 11;10(1):140. doi: 10.3390/microorganisms10010140 (PMC8777967; doi:10.3390/microorganisms10010140)
Supplement: Supplementary file 1 [file microorganisms-10-00140-s001.zip › microorganisms-1531060-supplementary.pdf]

Table S1. Mantel tests for correlations of microbial functional genes with between soil chemical properties or , biochemical activity and functional gene structure based on Bray-Curtis distance in untreated soils (C) and Salix treated soils (SRC) . * indicates *p*-value ≤ 0.05; ** = signifcant at *p*-value ≤ 0.01; *** = signifcant at *p*-value ≤ 0.001.

	C		SRC		Total (C&SRC)	
	r	p	r	p	r	p
Arylesterase	0.083	0.188	-0.153	0.917	-0.046	0.624
Arylsulfatase	-0.069	0.598	0.042	0.294	-0.130	0.942
Acid.phosphatase	0.065	0.238	0.178	0.096	0.113	0.091
Alkaline.phosphatase	0.091	0.196	-0.076	0.691	-0.101	0.939
phosphodiesterase	0.076	0.243	0.242	0.068	0.022	0.334
b-glucosidase	0.205	0.119	0.147	0.140	-0.077	0.793
b-galactosidase	0.119	0.129	**0.262**	**0.034***	0.088	0.145
urease	0.029	0.345	-0.020	0.421	-0.062	0.675
protease	-0.142	0.936	0.078	0.229	**0.212**	**0.001*****
dehydrogenase	-0.120	0.758	0.171	0.106	-0.024	0.562
ATP	-0.038	0.595	-0.050	0.647	0.080	0.158
CO2	0.133	0.145	-0.168	0.918	**0.264**	**0.027***
BioTox	0.018	0.351	**0.297**	**0.041***	-0.005	0.473
Nitrification potential	**0.353**	**0.025***	0.011	0.372	**0.238**	**0.016***
Ammonification potential	-0.023	0.532	0.023	0.372	0.056	0.262

pH	-0.060	0.632	**0.466**	**0.003****	0.071	0.170
Organic C	-0.061	0.562	-0.025	0.534	-0.080	0.784
Total N	-0.053	0.568	-0.159	0.903	0.011	0.389
NH4-N	0.113	0.165	0.030	0.320	0.103	0.128
NO3-N	-0.025	0.461	-0.052	0.568	**0.252**	**0.041***
Total P	**0.315**	**0.035***	-0.171	0.897	-0.104	0.846
Olsen P	-0.076	0.720	-0.002	0.466	**0.276**	**0.001*****
Soluble P	**0.380**	**0.002***	0.191	0.073	**0.178**	**0.008****
Organic P	-0.142	0.865	0.120	0.168	-0.034	0.525
Al tot	-0.049	0.589	0.067	0.207	0.136	0.090
As tot	-0.190	0.937	-0.043	0.595	**0.235**	**0.001*****
Cd tot	**0.246**	**0.017***	0.157	0.175	**0.326**	**0.001*****
Cr tot	0.183	0.094	-0.099	0.765	**0.203**	**0.001*****
Cu tot	0.102	0.207	-0.071	0.589	**0.239**	**0.001*****
Mn tot	0.236	0.088	0.075	0.214	**0.287**	**0.001*****
Ni tot	0.231	0.088	-0.030	0.547	0.144	0.064
Pb tot	0.048	0.298	-0.093	0.777	0.016	0.373
Zn tot	0.129	0.161	**0.290**	**0.044***	**0.204**	**0.021***
Na tot	-0.170	0.904	0.183	0.093	**0.241**	**0.001*****
Ca tot	0.078	0.202	0.096	0.139	**0.219**	**0.025***
K tot	**0.286**	**0.033***	0.129	0.143	**0.270**	**0.001*****
Mg tot	-0.111	0.913	0.130	0.161	-0.070	0.828
Fe tot	-0 056	0 518	-0 020	0 433	-0 067	0 705
Al av	-0 152	0 881	-0 028	0 455	**0 255**	**0 001*****
As av	-0 213	0 966	0 147	0 129	0 165	**0 047***
Cd av	0 037	0 312	0 041	0 303	**0 216**	**0 014***

Cr av	-0 006	0 449	-0 168	0 966	-0 004	0 452
Cu av	-0 123	0 806	-0 094	0 769	**0 230**	**0 001*** **
Mn av	-0 059	0 695	0 029	0 329	**0 158**	**0 024***
Ni av	0 200	0 134	**0 255**	**0 04***	0 056	0 169
Pb av	0 210	0 110	**0 231**	**0 04***	**0 259**	**0 001***
Zn av	-0 167	0 878	0 086	0 200	**0 149**	**0 035***
Na av	0 173	0 139	-0 006	0 455	-0 054	0 718
Ca av	-0 100	0 646	0 103	0 152	**0 134**	**0 038***
K av	-0 081	0 765	-0 140	0 914	0 023	0 329
Mg av	0 007	0 389	0 105	0 140	**0 175**	**0 001***
Fe av	-0 101	0 691	-0 019	0 473	**0 175**	**0 028***
